# Quality control in public participation assessments of water quality: the OPAL Water Survey

**DOI:** 10.1186/s12898-016-0063-2

**Published:** 2016-07-22

**Authors:** N L. Rose, S. D. Turner, B. Goldsmith, L. Gosling, T. A. Davidson

**Affiliations:** 1 Environmental Change Research Centre, Department of Geography, University College London, Gower St, London, WC1E 6BT UK; 2 Centre for Environmental Policy, Imperial College London, 13-15 Prince’s Gardens, London, SW7 1NA UK; 3 Department of Bioscience, Aarhus University, Vejlsøvej 25, Silkeborg, Denmark

**Keywords:** Citizen science, Open Air Laboratories, Quality assurance, Water quality, Water survey

## Abstract

**Background:**

Public participation in scientific data collection is a rapidly expanding field. In water quality surveys, the involvement of the public, usually as trained volunteers, generally includes the identification of aquatic invertebrates to a broad taxonomic level. However, quality assurance is often not addressed and remains a key concern for the acceptance of publicly-generated water quality data. The Open Air Laboratories (OPAL) Water Survey, launched in May 2010, aimed to encourage interest and participation in water science by developing a ‘low-barrier-to-entry’ water quality survey. During 2010, over 3000 participant-selected lakes and ponds were surveyed making this the largest public participation lake and pond survey undertaken to date in the UK. But the OPAL approach of using untrained volunteers and largely anonymous data submission exacerbates quality control concerns. A number of approaches were used in order to address data quality issues including: sensitivity analysis to determine differences due to operator, sampling effort and duration; direct comparisons of identification between participants and experienced scientists; the use of a self-assessment identification quiz; the use of multiple participant surveys to assess data variability at single sites over short periods of time; comparison of survey techniques with other measurement variables and with other metrics generally considered more accurate. These quality control approaches were then used to screen the OPAL Water Survey data to generate a more robust dataset.

**Results:**

The OPAL Water Survey results provide a regional and national assessment of water quality as well as a first national picture of water clarity (as suspended solids concentrations). Less than 10 % of lakes and ponds surveyed were ‘poor’ quality while 26.8 % were in the highest water quality band.

**Conclusions:**

It is likely that there will always be a question mark over untrained volunteer generated data simply because quality assurance is uncertain, regardless of any post hoc data analyses. Quality control at all stages, from survey design, identification tests, data submission and interpretation can all increase confidence such that useful data can be generated by public participants.

## Background

In aquatic science, and especially in water quality assessment, volunteer monitoring has been used for nearly 50 years. Lee [1] provides a history of volunteer water quality monitoring for the United States from its beginnings in the 1960s and the initiation of volunteer water clarity monitoring in Minnesota lakes in 1973 leading to the present annual ‘Secchi Dip-In’ where more than 2000 lakes nationally are monitored (http://www.secchidipin.org/index.html). In the UK, almost all public participation projects relating to freshwaters have been concerned with lotic water quality. In 1971, the Advisory Centre for Education (ACE), supported by a national newspaper, *The Sunday Times*, organised a river water quality survey for school children [2]. Nearly 5000 participants, mainly aged between 10 and 13, used a series of simple metrics including chemical tests and the identification of ‘indicator’ benthic macroinvertebrates to estimate the extent of water “pollution” across England and Wales. The received data was found to provide good agreement with that previously collected by professional biologists, but covered a greater geographical area. A similar exercise was undertaken between 1991 and 1993 with three annual surveys organised by Riverwatch and sponsored by National Power, the National Rivers Authority and The Wildlife Trusts. The first of these surveys asked participants to provide a description of the site, an assessment of the aquatic biota within the river (benthic invertebrate survey; fish information from anglers; aquatic plants) and simple chemical tests for nitrate, pH and carbonate. Data from the 500 responses were compared directly with the ACE survey from 20 years previously to show how water quality had improved or deteriorated in rivers at a regional scale [3]. Since then, such large-scale river and stream surveys have not been repeated although the Riverfly Partnership’s Riverfly Monitoring Initiative launched nationally in 2007, uses trained volunteer anglers to assess water quality on a monthly basis using estimates of caddisfly, mayfly, stonefly and *Gammarus* abundance (http://www.riverflies.org/rp-riverfly-monitoring-initiative). By contrast, no similar large-scale water quality surveys of standing waters have been undertaken in the UK. The ‘National Pond Survey’ in 1989 surveyed 200 minimally-impacted ponds while the ‘Impacted Ponds Survey’ and the ‘Lowland Ponds Survey’, both in 1996 surveyed 350 and 150 sites respectively [4]. None of these surveys employed the public to generate data. Pond Conservation’s (now Freshwater Habitat Trust) annual “Big Pond Dip” launched in 2009 focuses on garden ponds and received data from 250 participants in its first year (J. Biggs, Freshwater Habitat Trust, pers. comm.). Hence, at the start of the OPAL project there was scope for a national lake and pond surveying programme.

The Open Air Laboratories (OPAL) programme was launched across England in 2007 with the aim of bringing scientists and communities together to observe and record the natural world in local neighbourhoods, and is now being expanded across the whole of the UK [5]. Participation is principally via national surveys used to assess changes to biodiversity, environmental degradation and climate change [6]. The programme, funded by the UK Big Lottery Fund, provides educational materials to aid these investigations [6]. One of OPAL’s primary objectives is to encourage and facilitate participation in science among people who might not otherwise have the opportunity, so while OPAL survey participation is national and for all ages and abilities, the focus is on urban areas and in particular, deprived communities [7]. This principal of inclusion requires that all OPAL activities are ‘low barrier to entry’ with no requirement for training except that included within the survey materials themselves. However, nine regional Community Scientists were available during 2010 to offer training and advice on the water survey to groups and individuals when requested. The OPAL Water Survey was launched in May 2010. As with all OPAL surveys (e.g., [8–10]), and some other public participation water surveys (e.g., ‘Waterwatch Victoria’) [11] there were dual objectives of education and generating useful data, here, specifically an assessment of lake and pond water quality.

Public participation has been widely used in monitoring water quality [12] but there is a widespread concern over quality assurance of volunteer generated data (e.g., [13–16]) and participant objectivity [17–20]. This seems to be poorly addressed in many surveys using trained volunteers [12, 21] but is exacerbated by the OPAL approach of using untrained volunteers and largely anonymous data submission. However, in essence, the problems associated with either professional or volunteer generated data are the same. Both are of little value if monitoring or surveying is undertaken the wrong way [22] and without quality assurance and quality control measures, only a proportion is likely to be useable [23]. Appropriate tools therefore need to be in place to allow participants to produce data of known quality as well as helping users to extract useful information [15].

A number of recent papers describe the stages and requirements for constructing a successful public participation programme (e.g., [24–27]) but the scientific value of using simplified methods within these has been little studied [10]. The aim of this paper is to consider how quality assurance can be addressed in a public participation water quality survey especially where that involves untrained volunteers. We then apply these approaches to the OPAL Water Survey responses from 2010 to assess the extent to which data generated may be useable as a scientific dataset.

## The OPAL Water Survey

The main objective of the OPAL Water Survey was to gain a national ‘snap-shot’ assessment of water quality for as many lakes and ponds across England as possible. The use of public participation allowed access to many more lakes and ponds than would be possible by traditional monitoring programmes [24, 28], including some in private grounds that had never been surveyed before. To this end, a water survey ‘pack’ was compiled that included a series of activities and materials with the aim of providing something of interest to as many people as possible whilst generating useful data [5]. 40,000 packs were printed and freely distributed. All materials were (and remain) freely available to be downloaded from the OPAL website (http://www.opalexplorenature.org/WaterSurvey). Both biological and non-biological assessments were included in order to stress the importance of considering lakes and ponds in an holistic way. As with all OPAL survey materials, the OPAL Water Survey activities were pilot tested with ‘naive audiences’ [25] in order to ensure clarity of the step-by-step approaches and survey forms. Such an approach is crucial for untrained volunteer surveys. All instructions and protocols for undertaking the survey activities and submitting information (either directly online or Freepost return of paper copies for participants who had no internet access) were present within the pack. Once data had been entered onto the OPAL website, anyone with internet access was able to interrogate and explore all submitted data using a variety of online tools, data mapping and visualization techniques.

The OPAL Water Survey comprised four activities. Participants could take part in as many or few of these as they wished:

### An assessment of water quality using the presence and absence of broad, and easily identifiable, classes of aquatic invertebrate

The use of indicator species and freshwater communities to assess water quality has been in use for over 100 years ([29, 30] and references therein). Benthic macroinvertebrates are the most commonly used organisms for biomonitoring [31] with over 50 different macroinvertebrate-based assessment methods currently in use [32]. Aquatic macroinvertebrates are generally localised so their response to any stress is related to local conditions, they live for a period sufficient to identify impacts and display a wide range of sensitivity to water quality. They are also found in even the smallest water bodies, and are relatively easy to sample and identify to a broad classification level. These latter qualities in particular make the use of these organisms well suited to public involvement studies [33] and especially with school children [2, 34] while their use also avoids the need for the equipment required for equivalent chemical determinations [35].

Generating indices of water quality or scales of pollution from macroinvertebrate assemblages in streams has also been used for many years, from indices of general pollution or disturbance such as the Trent Biotic Index [36] and the Chandler Biotic Score [37] to more specific indices such as Hilsenhoff’s ‘Family level Biotic Index’ (FBI) for organic pollution [38] and the multi-metric ‘Benthic Index of Biotic Integrity’ (B-IBI) [39]. Many of these indices use a three-category tiered system for classifying degradation either as indicator tolerance classes of the invertebrate groups that are compiled to create the index (e.g., [21, 31, 40, 41]) or as a means to classify the scale of degradation of the stream itself [39].

The OPAL Water Survey used a similar approach, classifying aquatic invertebrates into 13 broad taxonomic classes, to each of which was allocated a ‘health score’ using a three-tiered system based on the invertebrate group’s tolerance to a broad range of stressors (Table 1). This system was based on the classification used by Pond Conservation (now Freshwater Habitats Trust) in their 2009 Big Pond Dip, itself developed from methods used by the National Pond Survey and the Predictive System for Multimetrics (PSYM) [42]. Invertebrates collected were identified into broad taxonomic groups using a four-page fold-out guide which included photographs, size guides and bullet-point key identification features for each. Health scores relating to the presence of each identified group (Table 1) were then summed to obtain a total ‘Pond health score’ for the lake or pond. A maximum score of 78 was therefore obtainable if all 13 taxonomic groups were found. ‘Pond health scores’ were then allocated into three classes also using the ‘Big Pond Dip’ system: Very healthy (score ≥31); Quite healthy (6–30) and Poor (or ‘Could be improved’) (0–5). A classification of a pond to ‘quite healthy’ therefore required the presence of at least one medium-sensitivity (5 score) invertebrate class while a ‘very healthy’ classification required the presence of at least one high-sensitivity (10 score) class (Table 1).

**Table 1:** OPAL Water Survey invertebrate classification and ‘Invertebrate group health’ score based on the tolerance of the group to a range of stressors. Below is a comparison of descriptors for the derived pond health score ranges for the OPAL Water Survey (2010) and the 2014 Big Pond Dip [54]

Tolerance class	Groups	Group health score
High sensitivity	Cased caddisfly larvae; Caseless caddisfly larvae; Dragonfly larvae; Damselfly larvae; Alderfly larvae	10
Medium sensitivity	Mayfly larvae; Water beetles (adults and larvae); Water bugs (including water boatmen, water scorpions, water stick insects etc.,); Pond skaters; Water shrimps	5
Low sensitivity	Water slaters (water hoglice); Worm-like animals (including chironomid larvae; flatworms; leeches; worms etc.,); Water snails (spired; limpets; planorbids)	1

Pond health score	OPAL Water Survey (2010) description	Big Pond Dip (2014) description
0–5	Poor or ‘could be improved’	Not yet great
6–30	Quite healthy	Good
31 and above	Very healthy	Brilliant

The use of simple sampling protocols that are not too demanding [28] and identification to broad taxonomic groups, which avoid taxonomic jargon [13] and do not require significant training, are widely used in volunteer water surveys (e.g., [2, 31, 34, 40, 41]) in order to maximise participant numbers [25]. Simple, scientifically tested methodologies available to the public have been found to obtain unbiased samples and accurate classification into taxonomic classes [41]. These can provide comparable patterns to officially accepted modern monitoring methods [43] and agree well with professionally collected data [2]. While there are certain key taxonomic groups which volunteers should be able to identify (e.g., Ephemeroptera, Plecoptera, Trichoptera, chironmids, oligochaetes and isopods) [21], attempting greater taxonomic identification in the field with volunteers is considered likely to introduce excessive errors [31, 33]. Classification to the broad taxonomic groups used within the OPAL Water Survey may therefore be the most appropriate especially for un- or self-trained volunteers [31]. A balance is clearly required between the simplicity that allows untrained citizens to undertake water quality monitoring and the level of sophistication required to make the resulting data useful [44].

Engel and Voshel [31] suggest that common protocols based on the presence and absence of benthic invertebrates, identified to broad classification levels and divided into three pollution categories are likely to overrate ecological condition. This is in contrast to other volunteer program data (e.g., [41]) (and our own—see below) which indicate that volunteers preferentially tend to miss smaller types of invertebrate thereby underestimating ‘pond health’ or water quality. While under-estimation may be as ‘dangerous’ as over-estimation, especially where such an assessment may act as a trigger for possibly expensive further work, for a broad-scale national snap-shot such as the OPAL Water Survey, under-estimation is probably preferable as it provides a worst case scenario. Whatever the approach, an assessment of potential volunteer sampling bias, accuracy of invertebrate identification and sensitivity of the protocols to sampling location and individual effort are required in order to determine the value of generated data. This can only be achieved through quality assurance programmes applied to each public participation study.

### An assessment of water clarity

Water clarity is a fundamental determinand in limnology as it provides an assessment of turbidity or suspended material in the water column. This determines the extent of light penetration and hence the potential for primary productivity and macrophyte growth on the lake bed. As a result there are indirect effects on habitat availability for aquatic fauna, stability of littoral areas, nutrient availability and pollutant transport within lakes [45, 46]. In the OPAL Water Survey, water clarity was measured using a bespoke device, termed the “OPALometer”, which comprised a white disc with 12 OPAL symbols arranged around the edge shaded in specific percentile gradations from light grey (5 %) to black (100 %) [5]. Participants weighted the disc by taping a small coin to the reverse and then pushed it through the neck of an empty 2 L clear plastic drinks bottle. The bottle was filled with pond water to a pre-determined level to provide a standard depth of water column. The participant then looked through the neck of the bottle, counted and recorded the number of OPAL symbols they could see. This device was calibrated against standard empirical measures of water clarity such as the Secchi Disc [47] and laboratory measures of suspended matter concentration (see below). Such a simple, visual approach to the assessment of turbidity allowed mass participation in this activity and is considered to be of greater educative value than, for example, taking a reading from an electronic meter [11].

### The measurement of lake water pH

pH, the measure of hydrogen ion (H^+^) concentration, is also fundamental in limnology. It drives many chemical processes in both catchment soils and freshwaters and can also determine which biological organisms may exist or thrive within a water body. Because of this, it is a parameter that is widely included in public participation surveys, for example the World Water Monitoring Challenge (http://www.worldwatermonitoringday.org/); the International Year of Chemistry’s ‘pH of the Planet’ (http://my.rsc.org/globalexperiment); and the OPAL Soil Survey [8].

There are many ways to assess pH and all have their advantages and disadvantages for large-scale surveys. For the OPAL Water Survey, two cheap, commercially-produced pH strips were included in each of the 40,000 packs and additional strips were sent free of charge to participants upon request. These were Pehanon^®^ pH 4.5–9.0 test strips manufactured by Macherey–Nagel (Germany). They are considered especially appropriate for a range of coloured waters and so are particularly useful for a national survey where lakes with high concentrations of dissolved organic carbon (DOC) may also be included. The test strips have the same accuracy over the whole measurement range (±0.25 pH unit) as tested against standard acid and basic solutions. However, as natural freshwaters tend to be weakly buffered, colour development of the test strip can take several minutes. Therefore, reading the strip too soon could result in the under-reading of water pH (A.Herzig and J. Tomatzky; Macherey–Nagel, pers. comms.).

## Methods: quality control and calibration approaches

Many studies have shown that volunteer-based schemes can provide reliable data and unbiased results [8, 13, 14] but Schmeller et al. [48] suggest that the quality of data collected by volunteers is more likely determined by survey design, methodology and communication skills than by the involvement of the volunteers per se. It is therefore as important to investigate quality assurance within the OPAL Water Survey methodologies as that of the participant-generated data itself.

### Invertebrate sampling method: sensitivity analysis

The OPAL Water Survey protocols provided simple instructions on how to sample for aquatic invertebrates. It was suggested that participants should look in as many different habitats around the lake and pond as possible and that, in each sampling location, a “vigorous sweep” of the pond net for 15–20 s amongst plants or other habitats should be undertaken. It is not possible to quantify how closely a volunteer (or indeed a professional scientist) adheres to a stated method from the reported data [11] but it is possible to determine how changes in sampling approach can affect those results. To provide such an assessment we undertook a multiple sampling exercise at ten lakes.

**Fig. 1:**
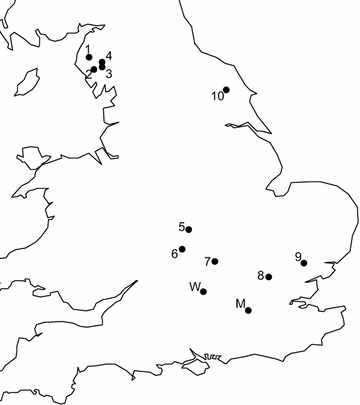
Site location map. Location map of multiple survey ponds: Marney’s Pond and Weston Green Pond (M); the participant experiment at the Little Wittenham Wood Pond (W) and the 10 calibration experiment lakes (*1*–*10*). *1* Loweswater, *2* Burnmoor Tarn, *3* Stickle Tarn, *4* Blea Tarn, *5* Combe Pool, *6* Compton Verney Lake, *7* Hydelane Lake, *8* Bonningtons Lake, *9* Preston’s Lake, *10* Scampston Park Lake

The lakes were selected to provide a broad cross-section of English standing water bodies for which the British Geological Survey already had archived data on catchment soil and inflow stream sediment chemistry to provide a calibration for the concurrent OPAL metals survey (http://www.opalexplorenature.org/metalssurvey). These lakes included three upland tarns with moorland catchments mostly used only for rough grazing and with largely homogenous, stony littoral zones, and six lowland lakes where catchments were more impacted (e.g., agriculture, dwellings) and where littoral habitats varied more widely including areas of emergent and floating macrophytes, and areas shaded by overhanging trees. Loweswater, in the Lake District, fell somewhere between these two site-types possessing a more agricultural catchment than the moorland Blea, Stickle and Burnmoor Tarns. Locations of these lakes are shown in Fig. 1 (sites 1–10) with site details provided in Table 2.

**Table 2:** Site details for the ten calibration experiment lakes

Site	Latitude	Longitude	Altitude (m a.s.l)	Lake area (ha)	Max. recorded depth (m)
Loweswater	54°34′52″N	03°21′19″W	125	60.3	16.5
Blea Tarn	54°31′02″N	03°05′44″W	478	7.4	12.0
Stickle Tarn	54°27′28″N	03°06′16″W	473	7.4	12.5
Burnmoor Tarn	54°25′46″N	03°15′28″W	253	23.9	13.0
Scampston Park Lake	54°09′53″N	00°40′27″W	32	4.9	1.2
Coombe Pool	52°24′43″N	01°25′15″W	73	30.6	2.0
Compton Verney Lake	52°10′07″N	01°33′04″W	78	13.1	2.0
Hydelane Lake	52°00′37″N	00°56′40″W	74	11.4	2.5
Preston’s Lake	51°57′22″N	00°41′51″E	51	7.7	3.5
Bonnington’s Lake	51°47′59″N	00°42′37″E	57	2.8	1.8

At each of these ten lakes, ten locations were selected around the perimeter and at each of these locations, three experienced surveyors each undertook a 10-, a 20- and a 30-s net sweep. This resulted in 90 invertebrate records for each lake except at Blea Tarn where only two surveyors were available (N = 60). As the surveyors worked together, surveys at each location were undertaken at the same time of day, precluding the influence of any diurnal changes. These surveys allowed an assessment of the effect on pond health score by individual sampling effort; on the variability of data generated by different individuals at the same location and time; and the effect of sampling at multiple locations around a lake compared to (and between) any one single location.

#### Effect of sampling effort

**Fig. 2:**
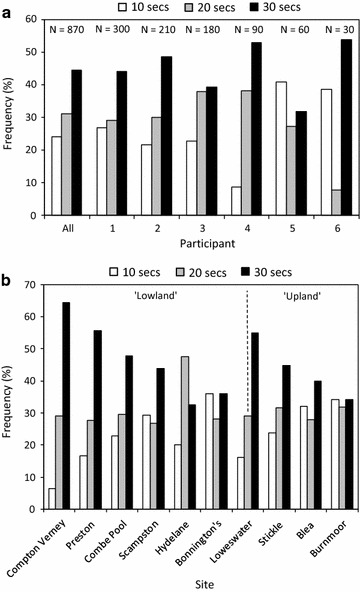
Effect of operator and sample time on ‘Pond health score’. **a** Frequency of highest ‘Pond health score’ for individual operators, and all operators combined, related to sampling effort (10, 20 and 30 s net sweeps). **b** Frequency of highest ‘Pond health score’ for all operators combined for each sampling site, related to sampling effort (10, 20 and 30 s net sweeps)

For the 870 surveys undertaken (all sampling sites; all surveyors; all sweep times), the highest ‘Pond health’ scores increased with sampling duration. 24.2 % of the highest scores were obtained by the 10 s sweep, 31.2 % by the 20 s sweep and 44.6 % by the 30 s sweep. Only the difference between 10 and 30 s sweep was significant at the p < 0.01 level (N = 290). Of the six individual surveyors, four had similar results to these overall scores (i.e., increased score with effort) while the frequency of the scores from the other two (who undertook the fewest surveys) varied more widely between the different sweep times (Fig. 2a). Only one surveyor returned any significant difference (p < 0.01) between sweep times, again between the 10 and 30 s sweeps (N = 30).

The same was also observed on a site-by-site basis (Fig. 2b). For seven of the ten lakes, highest scores were observed with the 30 s sweep (all sampling locations around the lake; all surveyors). The 20 s sweep produced the greatest frequency of high scores at Hydelane Reservoir, while at Bonnington’s Lake and Burnmoor Tarn, all three sweep times produced very similar frequencies of highest score. The greatest differences between sweep times appear to be at the lowland sites Compton Verney, Preston’s Lake and Combe Pool but also at Loweswater, and this may be due to greater habitat diversity around these sites. However, only the differences between 10 and 30 s sweeps (all surveyors) at Preston’s Lake and Compton Verney were significant (p < 0.01).

#### Effect of individual surveyors

**Fig. 3:**
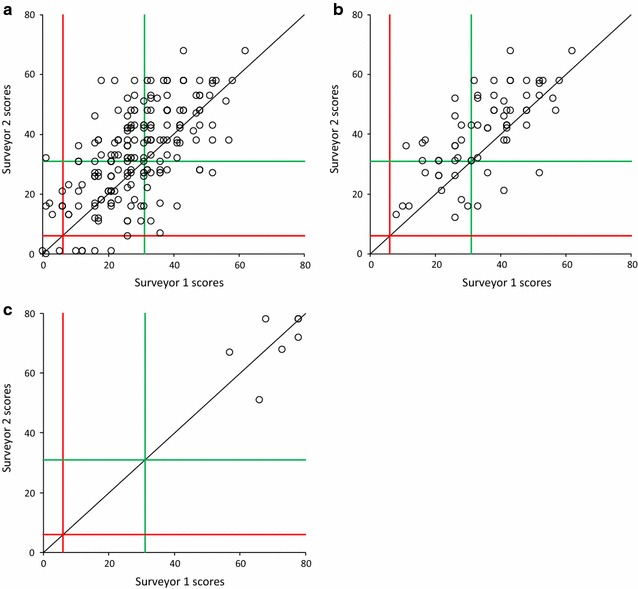
Comparison between pond health scores for two experienced surveyors. **a** At all lakes, all sampling locations, all sweep times (N = 210); **b** all lakes; all sampling locations within a lake (N = 70); **c** all lakes (N = 7). *Red* and *green lines* represent the poor and good thresholds for OPAL Water Survey health scores respectively. Hence, scores within the *boxes along the diagonal* represent surveyor ‘agreement’ within the OPAL three-tier system

This dataset may also be used to determine the differences between individual surveyors at the same sampling locations using the same sweep times. Considering the three surveyors who undertook the most simultaneous surveys (1–3 in Fig. 2a), significant differences (p < 0.01) exist between two of the three combinations of pairs. Given this level of variability between experienced surveyors, at least a similar level of variation might be expected between untrained OPAL Water Survey participants (see ‘multiple participant surveys’ below). However, while a certain level of variability between individuals is to be expected it is important to consider how these data are submitted and used within OPAL. As described above, the OPAL Water Survey protocol states that sampling should be in as many different habitats as possible around a pond and that, in each sampling location, a 15–20 s sweep should be undertaken. These data are submitted to the OPAL website at the ‘whole pond’ level and allocated to one of three tiers of water quality. Figure 3 shows how this approach affects individual survey scores. Figure 3a shows all the individual samples (all lakes; all sampling locations; all times) for one of our pairs of experienced surveyors. While there is obviously a considerable amount of scatter, the agreement between the two surveyors is reasonable (r^2^ = 0.39; p < 0.001; N = 210) and when water quality tiers are compared there is a 74 % agreement. If these individual samples are then amalgamated within a sampling location around a lake (i.e., 10, 20 and 30 s sweeps amalgamated for each location) then while the significance of the relationship decreases (r^2^ = 0.46; p = 0.015; N = 70) the agreement within the water quality tier increases to 81 % (Fig. 3b). Furthermore, if these are then amalgamated to the whole pond level, (the level at which they would be reported to OPAL) then this agreement increases to 100 % even though the relationship between the scores from the individual surveyors is no longer significant at any level (r^2^ = 0.25; p = 0.86; N = 7) (Fig. 3c). While the variability between individual samples even for experienced participants is quite high, once amalgamated to the ‘whole pond’ level and allocated to a water quality band, agreement is very good.

### Invertebrate identification: participant vs. scientist comparison

While previous studies have determined that field sampling of invertebrates for water quality assessments by trained volunteers and professional scientists can result in statistically indistinguishable data, especially where taxonomic identification is at Family level or above [13, 14], the emphasis of these studies has been on the adequate training of the participant volunteers. Little work has been undertaken on equivalent assessments with untrained participants. Although Heiman [49] suggests that professionals should sample side-by-side with volunteers in order to make a comparison, the results from our own 10-lake calibration dataset shows considerable variability occurs even between experienced people. Therefore, in order to assess the identification skills of untrained participants (rather than that individual’s sampling ability) the scientist needs to work on the same collected samples.

Using an approach similar to that of Au et al. [43] to make this assessment, a group of eight untrained participants aged 17–18 years undertook the OPAL Water Survey at a pond in Little Wittenham Wood, Oxfordshire (Fig. 1; Site W). Each participant sampled the same pond following the OPAL Water Survey guide but without any other specific instructions. They collected and identified their individual samples but were allowed to work in pairs as OPAL safety protocols indicate water survey activities should never be undertaken alone. They used only the OPAL Water Survey guide for identification but were allowed to confer with each other as would undoubtedly happen during normal survey participation. Three participants had time to undertake a second sample resulting in 11 individual analyses. Each individual sample was then also studied by a member of the OPAL Water Survey team to make a direct comparison.

**Fig. 4:**
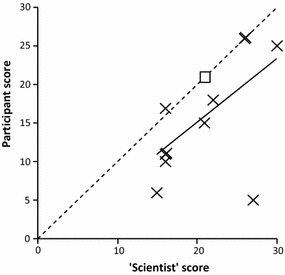
Comparison between untrained participant water survey scores and those of a member of the OPAL water team on the same sample. *Dotted line* is 1:1; the *open square* represents the sample undertaken by the member of the OPAL team. The *solid line* is the regression between the two sets of scores

In general, ‘water bugs’ (mainly corixids), mayfly larvae, snails and worm-like animals were all correctly identified. However, very small damselfly larvae were also present in a number of samples and these were often mis-identified as mayflies. This had the effect of reducing the derived pond health scores (Fig. 4) such that all except one sample lie below or on the 1:1 line. The participant group, although small, reflected a good cross-section of interest in aquatic environments and this appeared to be reflected in performance. The most enthusiastic participant undertook two samples, correctly identified all the invertebrates present in both and hence produced identical scores to the member of the OPAL team. By contrast, another less engaged participant only identified a single invertebrate group even though a number of others were present. This resulted in the largest discrepancy in the dataset. Without this latter score, the *r*^*2*^ value for this comparison was 0.83 (p < 0.01; N = 10) but including this score, the *r*^*2*^ dropped to 0.35 (p = 0.054; N = 11). This would suggest that in general invertebrate identification among untrained volunteers is reasonably good, especially amongst those motivated to participate by enthusiasm or interest in the activity.

These results agree very well with those of other studies (e.g., [34, 41]) who also reported that volunteers tended to miss smaller invertebrates in collected samples. This had the effect of reducing the numerical value of the derived metric (a biological integrity score) although these remained strongly correlated with professionally-derived data. Other studies have also indicated a lower taxonomic resolution in volunteer samples although performance is improved where additional aids (e.g., software keys; identification guides) are available [40, 50]. Greater experience in participation by undertaking further surveys would undoubtedly lead to more accurate identification (i.e., higher ‘observer quality’) [28].

### Invertebrate identification: self-assessment

The use of quizzes and games within participatory activities provides a tool by which to evaluate observer skill and determine a criterion for data inclusion [28]. Within the OPAL Water Survey, a short identification quiz was included at the end of the online data submission procedure. This multiple-choice quiz involved six pictures of aquatic invertebrates each with a number of possible identifications. Participants selected the name they considered correct and received a score out of six. It is to be assumed that as the participants had concluded the OPAL Water Survey they would be familiar with the provided identification guide and would probably have used this in undertaking the quiz. As this is presumably how the animals collected in their surveys were also identified, this was not considered a problem, but the quiz scores should be considered a ‘best available score’ as a result (cf. [40, 50]).

**Fig. 5:**
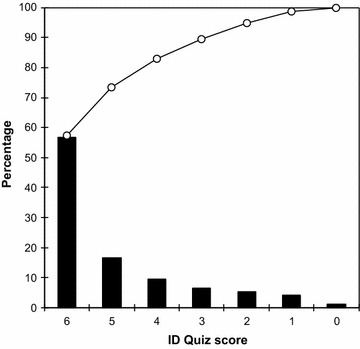
Results of the OPAL Water Survey invertebrate identification quiz undertaken during on-line data submission. Histogram shows the scores for the 2239 people who undertook the quiz as a percentage of the total. *Open symbols* show the cumulative percentage for each subsequent score

Figure 5 shows the results from 2239 participants who attempted the identification quiz while inputting OPAL Water Survey data online in 2010. These data show a sharp decline in success rate with 56.8 % of participants getting all six identifications correct, 16.7 % getting five correct, and declining through to 1.1 % who identified none of the invertebrate pictures correctly. Hence, by accepting (for example) only those data for participants who correctly identified five or six pictures, over 73 % of the data (1644 surveys) would remain for further analysis. Interestingly, the participants in the Wittenham Pond experiment, described above, all scored five or six correct identifications in this quiz. So, while using this criterion may lead to greater confidence in the dataset, it does not necessarily guarantee accurate identification within collected samples.

One further part of the online submission process allowed participants to add feedback about the survey, or additional information. These comments regularly showed participants’ own concerns over their identification ability, for example, not being “good enough for a scientific study”. Other feedback suggested that while they had enjoyed taking part in the activity, they didn’t submit their data for these same reasons. This anecdotal evidence indicates a general willingness for participants to try to identify the invertebrates to the best of their ability while the quiz provides a measure of how well they succeeded.

### Use of multiple participant surveys

**Fig. 6:**
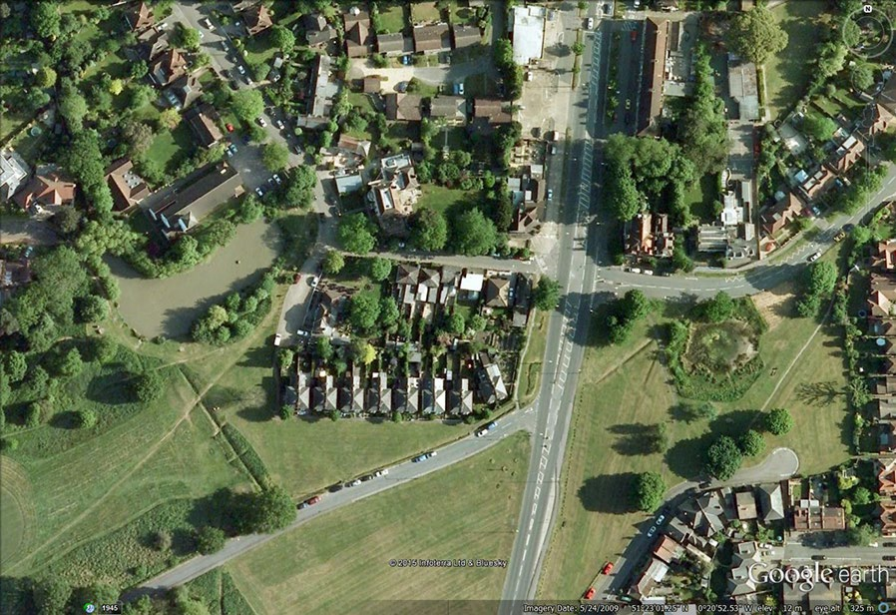
Aerial photograph of the two multiple-survey ponds. Google Earth image of Marney’s Pond (*left*) and Weston Green Pond (*right*) used for multiple OPAL Water Surveys by over 80 school children in July 2010. Image date 24th May 2009

On 6 July 2010 over 80 school children aged 10–11 each undertook the OPAL Water Survey on two ponds, Marney’s Pond and Weston Green Pond, both in Esher, Surrey (Fig. 1, site M). The surveys were undertaken within a short period of time and from a number of access points around each pond, although these were limited as the ponds are quite small (Fig. 6). Such ‘class activities’ allow an assessment of the variability in estimates of the different survey parameters. Here, these were recorded by a large number of individuals all with little experience either in invertebrate identification or in pH and water clarity measurement.

**Fig. 7:**
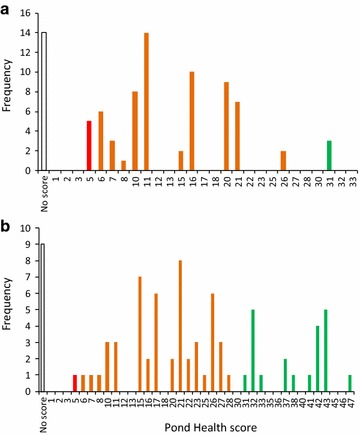
Pond health scores for the two multiple-survey ponds. Pond health scores for **a** Marney’s Pond and **b** Weston Green Pond as recorded by more than 80 school children on 6th July 2010. *Red*, *orange* and *green bars* denote ‘poor’, ‘medium’ and ‘high’ water quality respectively, as defined in the OPAL Water Survey

**Fig. 8:**
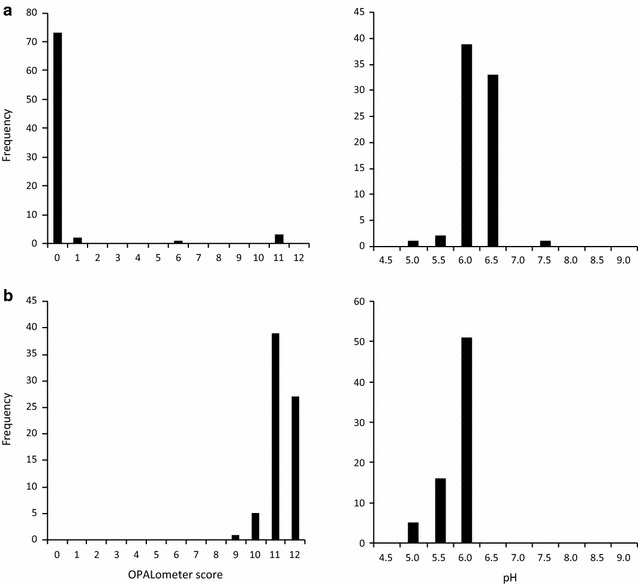
Water clarity and pH data for the two multiple-survey ponds. OPALometer and pH data for **a** Marney’s Pond and **b** Weston Green Pond as recorded by more than 80 school children on 6th July 2010

Figures 7 and 8 show the pond health scores, and pH and OPALometer data respectively, recorded for Marney’s Pond and Weston Green Pond (a and b). For both sites there is a broad distribution of pond health scores indicating considerable variability in the invertebrates recorded by the participants in their individual sample. Indeed, for both ponds all 13 invertebrate classes were recorded at least once providing a ‘theoretical health score’ for each pond of a maximum 78. However, no individual score got close to this maximum. For Marney’s Pond, individual scores tended to be lower and less distributed ( ${\bar{ \rm x}}$= 14.1; σ = 6.6; max = 31) than those at Weston Green Pond (${\bar{ \rm x}}$ = 24.3; σ = 10.8; max = 47). Our sensitivity analysis data (above) showed that experienced surveyors can also obtain quite variable scores for the same sampling location at the same time (Fig. 3a) and therefore this broad within-site variability may not be a ‘fault’ in participant sampling or identification, but rather sample variability, noise and, in this case, the possibility that the same location was sampled many times within a short period. To compensate for the first of these, the OPAL Water Survey allocates pond health scores to three ‘tiers’, reducing the emphasis placed on an individual score. Using this approach, of those participants providing invertebrate data for Marney’s Pond, 90 % produced values in the ‘quite healthy’ range of 6–30 (orange bars in Fig. 7a). Only eight participants produced other values and these were all close to the threshold (i.e., 5 and 31). For Weston Green Pond, of those participants recording invertebrate data, 70.8 % generated ‘quite healthy’ scores while a further 27.8 % derived higher ‘very healthy’ scores (Fig. 7b). Only one (1.4 %) recorded a ‘poor’ score of five. Hence, despite both ponds recording the same theoretical maximum these data would indicate both ponds are quite healthy, while the multiple participant data suggest Weston Green Pond may have a better water quality than that of Marney’s Pond.

The non-biological data generated by the children appear to show less variability. At Marney’s Pond over 92 % of the participants who returned water clarity data gave an OPALometer score of zero, while at Weston Green Pond over 91 % gave scores of 10 or 11 (Fig. 8). Verification of this distinction between the two ponds is clear from satellite images (Fig. 6) as are other catchment and pond characteristics recorded by the participants. By contrast, the pH data are similar as would be expected from two very closely located ponds (similar rainfall; geology; catchment characteristics). For Marney’s Pond over 94 % of participants recording pH data gave a value of 6.0–6.5, while for Weston Green Pond over 93 % recorded values 5.5–6.0, but predominantly (48.1 %) 6.0 (Fig. 8). Although these pH data are not independently verifiable they do show that the OPAL Water Survey approach does provide consistent results even among participants new to the techniques.

### Comparison with other variables

While participant data show good replicability for the non-biological parameters, there is a further question regarding how these data compare with more standard means of measurement. This is particularly important for the water clarity data as it can be used to provide a national picture of suspended solids in the water column.

**Fig. 9:**
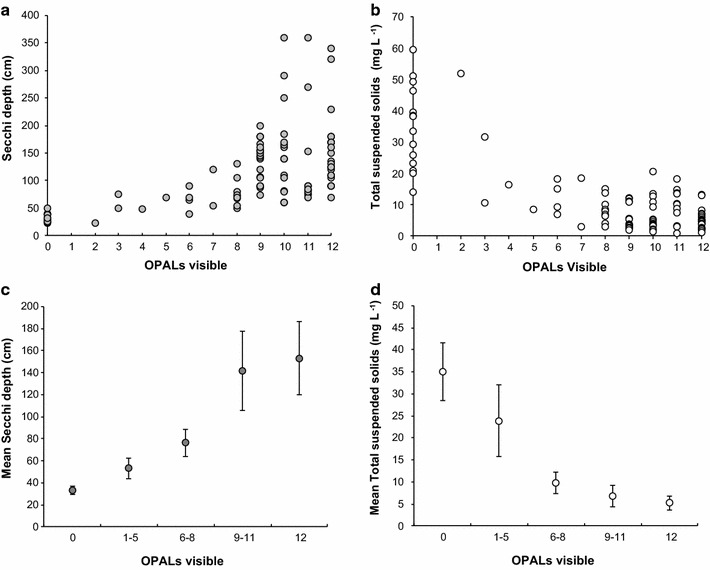
Comparison of water clarity data measurement techniques. Relationship between OPALometer readings and empirically measured **a** Secchi disc and **b** total suspended solids (TSS) from the OPAL water centre monitoring dataset [51] and conversion of OPAL scores into **c** Secchi disc depth and **d** total suspended solids concentration measurements

Figure 9 shows OPALometer data compared against both empirically measured Secchi disc depths and suspended solids measurements undertaken at nine lake sites monitored every 3 months over the course of the OPAL project [51]. There are good relationships between all these variables (r^2^ = 0.56 and 0.47 for OPALometer vs. Secchi depth and suspended solids respectively) as observed in previous water clarity comparisons between turbidity meter and turbidity tubes [11]. One limitation of the OPALometer is that suspended solids can increase beyond the point at which no OPAL logos are visible resulting in a broad range at this lowest value (Fig. 9b). Similarly, Secchi disc depth can also increase beyond the point at which all 12 OPAL logos are visible, again resulting in high variability at this point on the scale (Fig. 9a). Comparison between these measurement approaches therefore needs to be interpreted with caution at highest and lowest water clarities and this is in agreement with previous community-generated water clarity data which was found to be most inaccurate at highest and lowest clarity levels [11]. Consequently, we again used a three-tier approach to the intermediate OPALometer scores and calculated a relationship between these, and with both suspended solids concentrations and Secchi Disc depth (Fig. 9c, d), which could be applied to the national OPAL Water Survey data.

### Use of other metrics

**Fig. 10:**
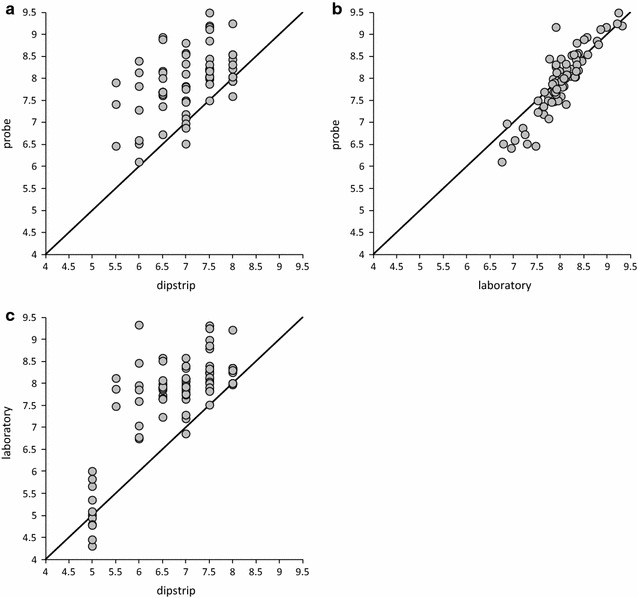
Comparison of pH data measurement techniques. Comparison of pH measurements between field probe and **a** dipstrip and **b** laboratory measurement. Also **c** the comparison between laboratory and dipstrip measurements. 1:1 lines are also shown

The pH dip-strips employed in the OPAL Water Survey may only realistically be expected to provide an indication of water pH but it is of interest to assess their performance against other, more standard, means of measurement in order to determine how these data may best be interpreted. Estimates of pH made using the dipstrips were compared simultaneously in the field with a calibrated pH probe (Hach HQ30d with PHC101 probe) and also against laboratory measurements on water samples collected at the same time (National Laboratory Service; automated electrode probe; RSD of 0.2, 0.5 % accuracy measured against pH 7.6 quality control standard, N = 398; NLS pers comm.). Figure 10 shows a comparison of these three methods. As expected, the field pH probe and laboratory measurements show very good agreement (r^2^ = 0.82; p < 0.01; N = 80; Fig. 10b) and largely plot along the 1:1 line although the field probe may slightly under-read with respect to the laboratory analysis. By contrast, the dip-strips under-read and appear to show a considerable range against both probe and laboratory measurements (Fig. 10a, c respectively). This may be at least partly due to insufficient time being given for dip-strip colour development in weakly-buffered natural waters. Including a further set of data from upland lakes with lower pHs in the comparison between dip-strips and laboratory measurements (Fig. 10c; no probe data available) appears to improve the relationship (r^2^ = 0.58; p < 0.01; N = 80) but this is undoubtedly driven by the lower pH values. While the dip-strips give an unchanged value of pH 5.0 there is considerable variability in laboratory pH measurement for the equivalent water samples (pH 4.45–6.0). Therefore, while the pH dip-strips employed in the OPAL Water Survey may provide a rudimentary field assessment of pH and undoubtedly allow participants to consider important non-biological parameters in lakes and ponds, this approach is unlikely to generate robust data.

### Summary of quality assurance

It is evident that there is considerable variation between individual samples taken from around a lake or pond even when sampling is undertaken by experienced surveyors. However, the approach used by the OPAL Water Survey, of using broad taxonomic classes, amalgamating multiple habitat surveys into a single ‘Pond health score’ and allocating that score into three tiers of water quality provides sufficient latitude for variations in individual sampling technique (sampling time; number of locations; individual effort) and, to some extent, inexperience in invertebrate identification. However, increasing discrepancy is to be expected moving through the continuum of sampling possibilities from a single, short sweep in one location at a lake through to a multi-habitat, multi-sample strategy. This will also be influenced by lake size and the availability of differing habitats at the site.

It would appear that most participants will try to undertake the survey to the best of their ability, and untrained volunteers, motivated to take part by enthusiasm and interest, generally appear to be concerned about data quality and some even decline to submit their data as a result. Untrained or inexperienced volunteers are most likely to miss, or mis-identify, smaller invertebrates resulting in lower pond health scores and providing an under-estimate for any generated data. They may also sample less efficiently than experienced participants thereby also potentially reducing their sample scores. Performance would undoubtedly improve with experience leading to greater ‘observer quality’ and hence more reliable data. Use of a self-assessment invertebrate identification quiz provides a means to make a broad judgement of a participant’s taxonomic skills and could be used to remove data associated with the lowest identification scores and increase confidence in the remaining dataset. However, this approach does not help with the possible reduced effectiveness of an inexperienced participant’s sampling technique.

**Fig. 11:**
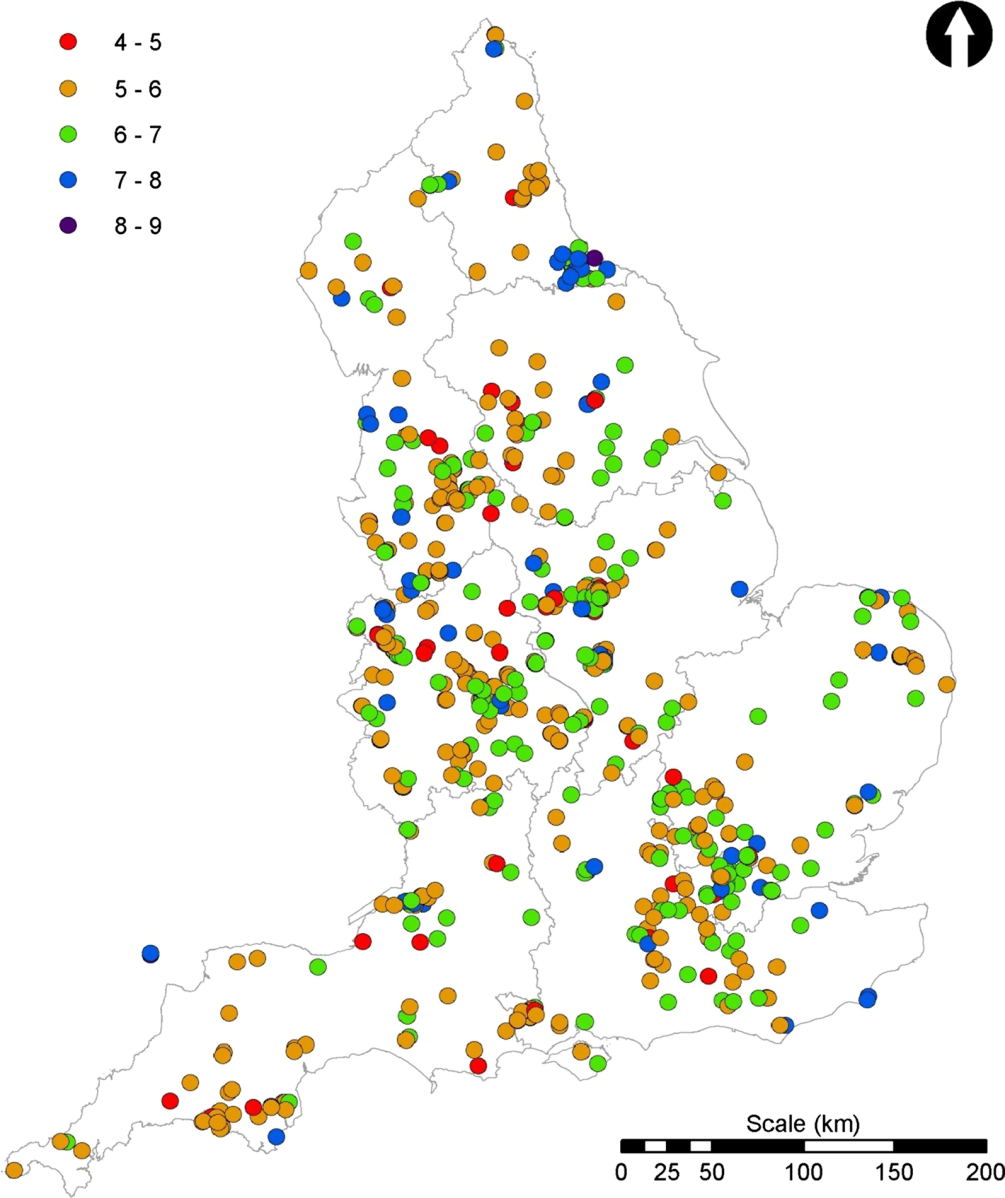
pH data for the 2010 OPAL Water Survey final dataset. The 1609 OPAL Water Survey sites included in the final dataset showing the reported pH data. The data points apparently located in the sea in the south–west are from ponds on Lundy island

Of the activities within the OPAL Water Survey, the invertebrate sampling is able to provide useful water quality scores while the water clarity results, when calibrated to empirical measurements, can generate broad-scale suspended solids data. By contrast, and despite the consistent data generated by the school children at Marney’s Pond and Weston Green Pond (Fig. 8) there appears to be considerable variation in the pH measurements in some waters depending on the time allowed for the colour to develop. This is likely due to the response of the pH strips in natural, low ionic-strength waters but also due to the survey instructions which did not provide sufficient emphasis on allowing time for the colour to develop. Although improving these instructions would help, it is likely that, while cheap and indicative of acid/base status to a broad level, within the context of public participation surveys, these pH strips may not be providing data of sufficient quality to interpret further. Here, we simply present the pH data as submitted (Fig. 11). In summary, with careful consideration of the data and some simple quality assessment we believe that untrained volunteers can provide useful water quality data.

## OPAL Water Survey results and discussion

### Participation

**Fig. 12:**
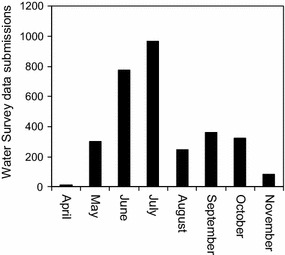
Data submissions for the OPAL Water Survey: April–November 2010

The OPAL Water Survey was launched 4 May 2010. Data continue to be submitted to the OPAL website and more than 4600 data entries had been entered by the end of 2013. A few surveys were submitted prior to the official launch date as a result of local training days for OPAL Community Scientists. Here, we focus only on the data submitted between April and November 2010 to remove any issues relating to inter-annual variability. During this period 3090 surveys were submitted online or returned as hard copies. Peak submissions occurred at the end of June and in July (Fig. 12) probably because this represents the last few weeks of the academic year when it was reported that many school groups took part.

For the first phase of OPAL from 2007 to 2013, England was divided into nine regions [6]. 27.9 % of the total surveys submitted in 2010 were undertaken within the West Midlands, 14.3, 12.5 and 10.9 % were undertaken in the southeast, northwest and southwest regions of England respectively. The remainder of the regions all returned <10 % of the total with London, the smallest region by area, submitting just 3.9 %. It is difficult to estimate the total number of people who took part as respondents did not include the number of participants for each survey. However, in addition to independent data returns, OPAL Community Scientists reported that they had worked with over 4900 people on the water survey in 2010. Of these, 15 % could be classified as ‘hard to reach’ and these included people from areas of deprivation, black and ethnic minority groups and people with disabilities [7]. In terms of educative value, 94.7 % of survey questionnaire respondents said they had learned something new from the OPAL Water Survey compared with 89.8 % for OPAL overall [7]. Four per cent of water surveys were carried out in areas in the top 10 % of most deprived areas in England [52].

### Initial data screening

**Fig. 13:**
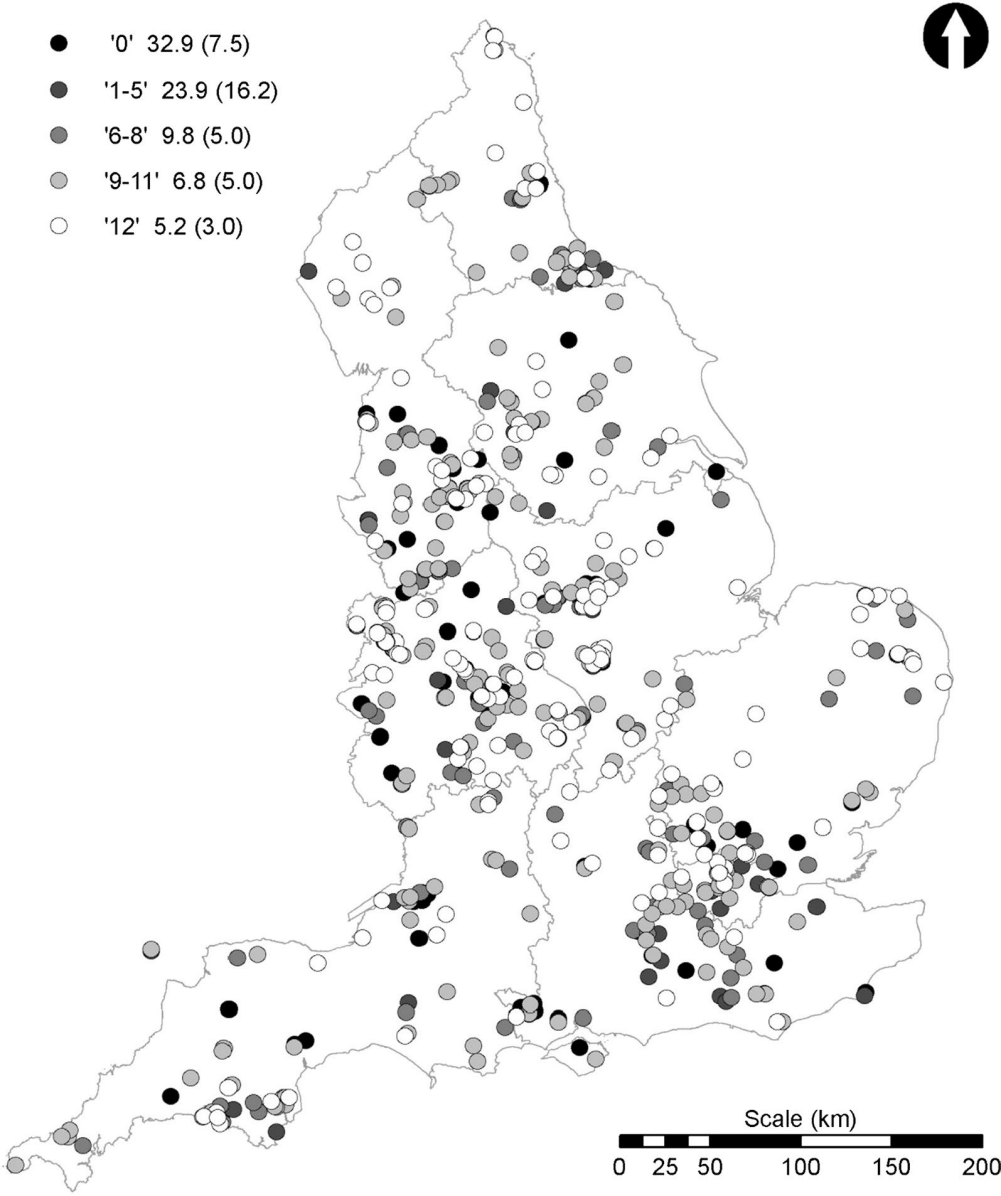
Distribution of total suspended solid (TSS) concentrations. *Scale* shows the OPALometer score range (e.g., ‘1–5’), and the mean and standard deviation (in *parentheses*) of the TSS concentration for each (mg L^−1^)

**Fig. 14:**
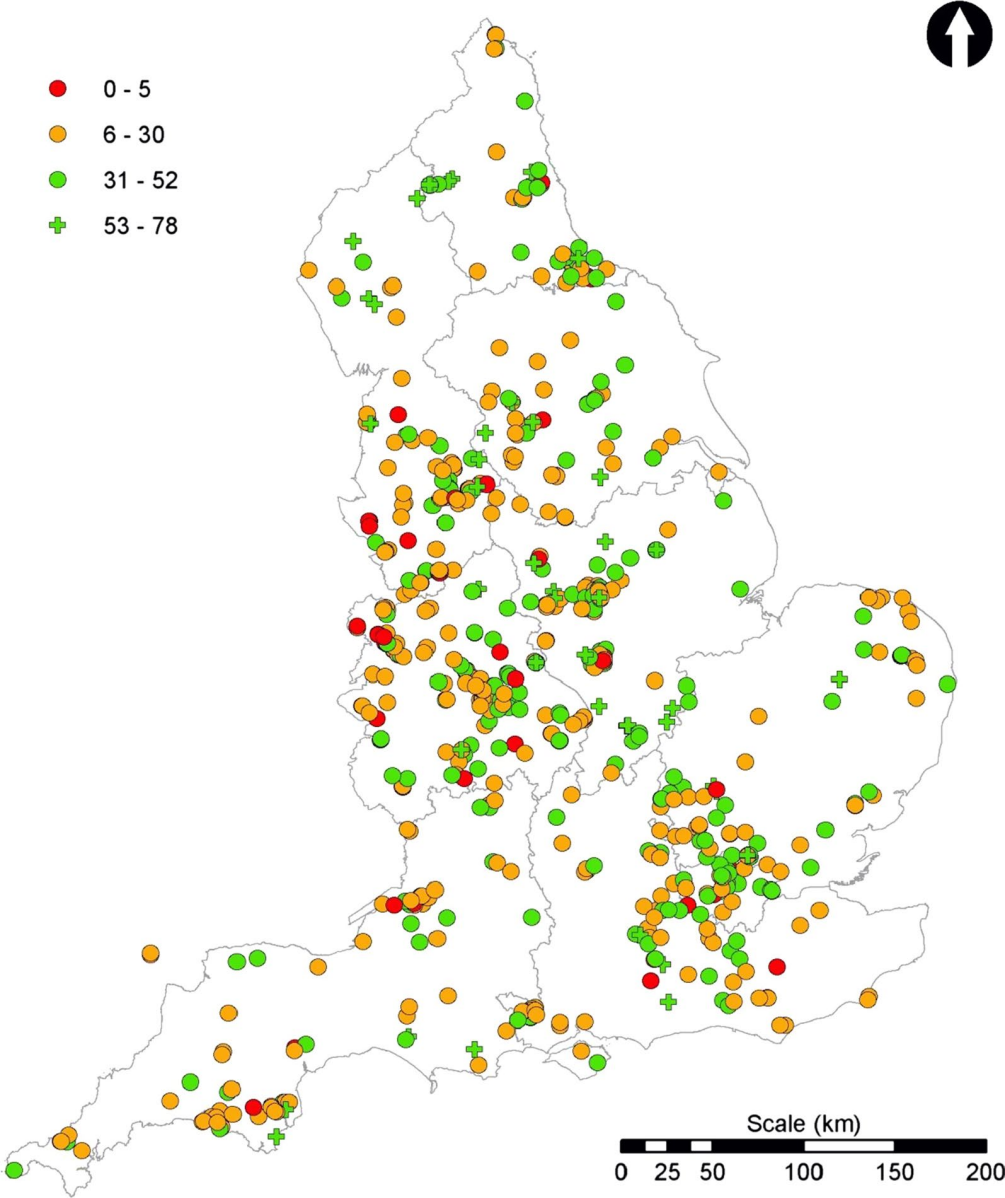
Water quality data for the 2010 OPAL Water Survey final dataset. Pond health scores derived from the invertebrate data are divided into the three water quality tiers and an additional ‘excellent category’ (53–78) (see text). The nine OPAL regions of England are also shown

The 3090 data submissions were initially ‘cleaned’ by removing sites outside England; sites providing locations which were in the sea; and those where no lake or pond was identifiable from a satellite image and where that location indicated a very low likelihood of a lake or pond being present or close by (e.g., roads, buildings). This resulted in 2897 data entries. Submissions without an invertebrate ID quiz score and those with quiz scores of less than five were then removed. This resulted in a final dataset of 1609 sites distributed across England (Figs. 11, 13, 14) and represented 52 % of the total surveys submitted in 2010. These 1609 sites included large lakes to garden ponds, and, from within each region, urban, sub-urban and rural sites.

### Water clarity

**Fig. 15:**
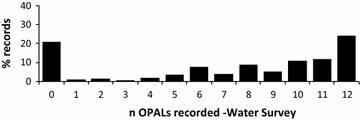
Breakdown of individual OPALometer scores recorded as a percentage of the 1546 participant submissions

Of the 1609 surveys, 63 (3.9 %) were submitted without an OPALometer score. The results from the remaining 1546 surveys were dominated by the two end members of visibility; none visible (zero OPAL logos, 21 %) and all visible (12 OPALs, 24 %) (Figs. 13, 15). High water clarity values dominate nationally (median = 10 OPALs) due to a significant number of surveys recording 10 (10.7 %) and 11 (11.6 %). This largely bi-modal distribution of OPALometer scores was also observed in the quarterly monitoring programme of nine lakes and ponds during the OPAL Water Centre monitoring project (n = 142) [51].

The mid-range OPALometer scores (2–8) indicate a Secchi depth <1.5 m (Fig. 9c). The range of Secchi depths between nine and 12 OPALs can be explained by data from upland lakes with higher dissolved organic carbon (DOC) content which are ‘transparent’ in the short water column depth of the OPALometer but give a shallow (<1.5 m) Secchi depth. The same effect was observed when algal blooms reduced the Secchi depth but had little effect on the number of OPALs observable.

Total Suspended Solids (TSS) vs. OPALometer scores were similarly affected. Between 1 and 5 OPALs, TSS values reduced significantly with improved water clarity (Fig. 9b) while an OPALometer score greater than 6 provides a TSS estimation of <20 mg L^−1^ (Fig. 9d). Viewed nationally, good and very good water clarity in ponds and lakes dominate (Fig. 13) but at a smaller scale the pattern becomes random and site specific. The spatial autocorrelation of water clarity is highly clustered (Moran’s I = 0.26, Z-score = 19.53) and this is caused by two factors. First, multiple measurements were taken at the same site(s) (e.g., school class exercises) and second, that the sampling of ponds and lakes did not occur systematically.

### Invertebrate-derived estimates of pond health

The pond health scores from the 1609 sites are presented in Fig. 14. Overall, 8.4 % of all sites showed ‘poor’ (or ‘could be improved’) water quality; 64.8 % were ‘quite healthy’ and 26.8 % were ‘very healthy’. All regions showed similar distributions to this national picture except East of England where the ‘quite healthy’ and ‘very healthy’ categories scored approximately equally and in East Midlands where the frequency of ‘very healthy’ lakes and ponds exceeded the ‘quite healthy’ ones (54.9: 41.5 %). In all regions the ‘poor’ category included only 1.4 % (northeast) to 9.8 % (West Midlands) of the total number of sites. Similar to the measurements of water clarity, the spatial autocorrelation of health scores is clustered (Moran’s I index = 0.3, Z-score = 22.9).

**Fig. 16:**
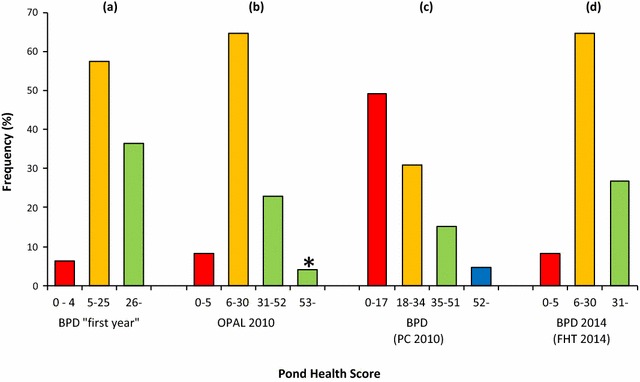
The effect of changing water quality tiers on the 2010 OPAL Water Survey final dataset. OPAL Water Survey 2010 pond health score dataset classified into water quality bands using **a** the original Big Pond Dip scheme [42]; **b** the OPAL Water Survey scheme with the additional “excellent” classification (marked as *asterisk*; see text); **c** the Big Pond Dip ‘equal bands’ scheme “for detailed interpretation” [42] and **d** the Big Pond Dip 2014 classification [54]

Health scores from all categories were present in both urban and rural sites in each region. Furthermore, in each region, some sites scored very highly indeed, despite concerns over the possibility for volunteer underscoring. In order to identify these highest quality sites an additional ‘excellent’ category was added where pond health score exceeded 52, requiring the presence of at least three classes of invertebrate from the highest sensitivity band. Sites in this category were present in every region (Fig. 14) but nationally included just the highest 4.0 % (* on Fig. 16b). However, no observed criteria were able to distinguish these from other lakes or ponds in the dataset.

Although the addition of this new ‘excellent’ category simply split the upper group into two (Fig. 16b), the alteration of classes during monitoring programmes should be avoided. In order to make meaningful and reliable comparisons there is a need for consistency not only by participants in sampling and identification but also in data assessment. The strength of monitoring lies in longevity and consistency of method, and public participation monitoring is no exception. Participants should be able to observe how, over time, water quality is improving or deteriorating, the extent to which any attempts at improvement have been successful, or conversely, how any new impacts have had deleterious effects. An example of how changing data assessment criteria could influence interpretation is presented in Fig. 16. This divides the 1609 pond health scores into water quality categories using approaches from recent UK pond surveys. Pond Conservation’s (now Freshwater Habitat Trust) ‘Big Pond Dip’ (BPD) launched in 2009 used a three-tier scheme with classifications of 0–4 (“could be improved”); 5–25 (“good”) and 26 and above (“really good”) [42]. The OPAL Water Survey classification was based upon this, with minor modification, and so the distributions between the first BPD and OPAL classifications are very similar especially when the OPAL ‘excellent’ category is included in the ‘very healthy’ class (Fig. 16). However, in 2010, Pond Conservation changed their classification to “four bands of equal width.. [to].. assist in interpretation” [42]. If this classification is applied to the 1609 scores a vast increase in the number of lakes and ponds allocated to the two poorest health classes results (i.e., 0–17 “low”; 18–34 “moderate”; Fig. 16c). This alters the frequency distribution of pond health scores and hence any interpretation that would stem from it. This may explain the apparently contradictory conclusions of BPD and OPAL whereby BPD 2009 concluded “about half of all ponds were in poorer condition” [53] whereas the OPAL data from 2010 show over 60 % were ‘quite healthy’ and a further 26 % were ‘very healthy’. In 2014, the BPD reverted to using the same classification scheme as the OPAL Water Survey with three tiers of 0–5 (“not yet great”), 6–30 (“good”) and 31 and above (“brilliant”) (Table 1) [54]. Applying these to the OPAL 2010 dataset (Fig. 16d) provides a similar frequency distribution to the original BPD and, of course, the OPAL Water Survey.

### Using volunteers for water quality monitoring

The OPAL Water Survey generated a wealth of data on a variety of ponds and lakes across England (and beyond) many of which had not been surveyed before. The high pond health scores reported for these sites show that both natural and artificial ponds, in rural and urban settings, can have a high aquatic diversity [55]. In particular, the value of these ponds lies in the varied habitats they can provide [56], highlighting the need to sample in as many habitats as possible for a more reliable and repeatable pond health score, particularly in lowland sites where lakes may have a greater number of habitat types.

While the requirement for multiple habitat sampling was stressed in the OPAL protocol it is not possible to tell from how far along the sampling continuum any particular datum was derived but it must be assumed that participants attempted the sampling programme to the best of their abilities. Anecdotal evidence from within OPAL would certainly suggest that this was the case. The data presented above shows that the OPAL approach allows a certain amount of latitude in sampling and that the simple identification and classification allows the generation of repeatable results especially where information from multiple habitats around a pond are amalgamated to a single pond health score. While such considerations make the ‘worst case’ presumption that no participants had undertaken similar exercises before, many will undoubtedly have been enthused to take part by having done surveys previously while the OPAL Water Survey experience will hopefully encourage others to undertake more in the future. Hence, over a longer term where participants undertake the monitoring of ‘their’ lake or pond on multiple occasions it would be expected that sampling reliability and identification will improve and therefore the data produced more robust. However, it is important to note that as a participant’s experience and skill improves they may also sample more efficiently and therefore find and identify more invertebrate groups than they would previously have done. Hence, scores might increase due an increase in sampling skill rather than because the pond water quality has improved.

The detection of broad-scale trends at multiple sites is a particular strength of volunteer monitoring. While the identification of invertebrates to broad classes may never be sensitive enough to detect subtle changes in water quality [21], a lower taxonomic level is sufficient to detect the impact of a perturbation on an aquatic community [33, 57]. Detection of trends is strengthened by the three-tier classification approach where at least one mid-sensitivity invertebrate class is required for the ‘quite healthy’ score while a ‘very healthy’ classification requires the presence of at least one high sensitivity invertebrate group. This is particularly important in surveys such as the OPAL Water Survey and the Big Pond Dip where the same pond health score could be generated by different combinations of invertebrates. This 3-tier approach therefore avoids potential confusions, for example where combinations of mid-sensitivity invertebrates could raise the pond health score to a higher health tier without the presence of high-sensitivity classes and may also help take account of variations due to sampling technique or dissimilarities between volunteer observations.

## Conclusions

To conclude, we return to the question of whether untrained volunteer participants can provide scientifically useful water quality data. It is likely that there will always be a question mark over such data simply because quality assurance is uncertain, regardless of any number of post hoc data analyses. This is exacerbated by the approach used by OPAL where data submissions can also be anonymous even though this was designed to increase participation number. In undertaking such surveys, there is a need to assume that participants have undertaken the activities using the described protocols to the best of their abilities. If this is the case, and the questions and techniques are simple and clearly explained, then there is no reason why these data should not be useful and available on a much greater spatial scale than would otherwise be possible.

There are means by which quality assurance can be improved in public participation water quality surveys. Training volunteers where possible (e.g., Freshwater Habitat Trust’s PondNet survey [58]); the use of repeat surveys to gain experience; re-surveying by experienced personnel; and the ability to provide feedback (although this requires non-anonymity) would all provide more confidence in data collection. Further, the inclusion of quality control at all stages, from survey design, identification tests, data submission and interpretation can also increase the confidence in a final dataset. As with all monitoring and survey work, either professional or voluntary, consistency of approach in sampling, interpretation and assessment of data are key, while experience through undertaking more surveys would also undoubtedly improve data quality even for initially untrained and anonymous volunteers. However, for projects such as OPAL, data collation is only one of the aims. A consideration of the benefits to education, raising environmental awareness and ecological literacy, and an appreciation of the natural world are also important.

## Abbreviations

ACE: Advisory Centre for Education
ARK: action for the river Kennet
B-IBI: Benthic Index of Biotic Integrity
BPD: Big Pond Dip
DOC: dissolved organic carbon
EPA: Environmental Protection Agency
FBI: Family-level Biotic Index
FHT: Freshwater Habitats Trust
ha: hectare
m: metre
m.a.s.l: metres above sea-level
NLS: National Laboratory Service
OPAL: Open Air Laboratories
PSYM: Predictive System for Multimetrics
TSS: total suspended solids
UK: United Kingdom
